# Prolactin and Family Psychiatric History in Schizophrenia

**DOI:** 10.1192/j.eurpsy.2023.947

**Published:** 2023-07-19

**Authors:** I. Bouguerra, E. Khelifa, A. Adouni, S. Sdiri, H. Abaza, H. Ben Ammar, L. Mnif

**Affiliations:** 1 F pyshciatry departement; 2 Razi Hospital, Mannouba, Tunisia

## Abstract

**Introduction:**

Schizophrenia is a chronic and multifactorial mental disorder. Research suggests the presence of an abnormality in prolactin secretion during the genesis of the disease and at the same time, the involvement of genetics in its pathogenesis has long been the demand of researchers in the field of genetics since familial forms of schizophrenia have been observed.

**Objectives:**

The objective of this study was to describe the prolactin profile and and to study its relationship to the patients’ family history of pyshciatric illness.

**Methods:**

This was a descriptive, cross-sectional study of thirty male patients hospitalized for a psychotic relapse who were naïve or discontinuing treatment for at least two months. Patients were assessed using a semi-structured questionnaire. A blood sample was taken to measure levels of prolactin.

**Results:**

The age ranged from 17 to 56 years. Most patients had a family medical history. Twenty patients (66%) had a family psychiatric history of schizophrenia (56%), mental retardation (3%), personality disorder (3%) and schizoaffective disorder (3%). Prolactin levels ranged from 0.5 to 45.67 ng/mL with a mean of 14.03 ng/mL. Seven patients (23%) had hyperprolactinaemia. All patients with hyperprolactinaemia had a family history of psychiatry with a statistically significant difference (p= 0.033).

**Conclusions:**

Hyperprolactinemia could be one of the “endophenotypes” that reflect a vulnerability to schizophrenia, found in familial forms of the disease. In this context, longitudinal studies on a larger scale and family studies including siblings without schizophrenia should be undertaken.

**Disclosure of Interest:**

None Declared

